# Novel transcripts from a distinct promoter that encode the full-length AKT1 in human breast cancer cells

**DOI:** 10.1186/1471-2407-14-195

**Published:** 2014-03-15

**Authors:** Jeffrey W Schmidt, Barbara L Wehde, Kazuhito Sakamoto, Aleata A Triplett, William W West, Kay-Uwe Wagner

**Affiliations:** 1 Eppley Institute for Research in Cancer and Allied Diseases, University of Nebraska Medical Center, 985950 Nebraska Medical Center, Omaha, NE 68198-5950, U.S.A; 2 Department of Pathology and Microbiology, University of Nebraska Medical Center, 985950 Nebraska Medical Center, Omaha, NE 68198-5950, U.S.A

**Keywords:** Human, Mice, Transgenic, Breast cancer, Mammary cancer, Proto-oncogene protein c-akt, Gene expression mRNA

## Abstract

**Background:**

The serine-threonine kinase AKT1 plays essential roles during normal mammary gland development as well as the initiation and progression of breast cancer. *AKT1* is generally considered a ubiquitously expressed gene, and its persistent activation is transcriptionally controlled by regulatory elements characteristic of housekeeping gene promoters. We recently identified a novel *Akt1* transcript in mice (*Akt1m*), which is induced by growth factors and their signal transducers of transcription from a previously unknown promoter. The purpose of this study was to examine whether normal and neoplastic human breast epithelial cells express an orthologous *AKT1m* transcript and whether its expression is deregulated in cancer cells.

**Methods:**

Initial sequence analyses were performed using the UCSC Genome Browser and GenBank to assess the potential occurrence of an *AKT1m* transcript variant in human cells and to identify conserved promoter sequences that are orthologous to the murine *Akt1m*. Quantitative RT-PCR was used to determine the transcriptional activation of *AKT1m* in mouse mammary tumors as well as 41 normal and neoplastic human breast epithelial cell lines and selected primary breast cancers.

**Results:**

We identified four new *AKT1* transcript variants in human breast cancer cells that are orthologous to the murine *Akt1m* and that encode the full-length kinase*.* These transcripts originate from an alternative promoter that is conserved between humans and mice. *Akt1m* is upregulated in the majority of luminal-type and basal-type mammary cancers in four different genetically engineered mouse models. Similarly, a subset of human breast cancer cell lines and primary breast cancers exhibited a higher expression of orthologous *AKT1m* transcripts.

**Conclusions:**

The existence of an alternative promoter that drives the expression of the unique *AKT1m* transcript may provide a mechanism by which the levels of AKT1 can be temporally and spatially regulated at particular physiological states, such as cancer, where a heightened activity of this kinase is required.

## Background

The PI3-kinase/AKT pathway is one of the most frequently altered signaling cascades in a large variety of human cancers. The deregulated expression and activation of signal transducers in this pathway can occur through various mechanisms such as hereditary or sporadic mutations (e.g., *PIK3CA*, *PIK3R1*, *AKT1/3, PDK1*), amplification or transcriptional upregulation (e.g., *PIK3CA*, *AKT1/3*), transcriptional repression or deletion of negative regulators such as *PTEN*, as well as increased expression or activity of growth factors and their corresponding receptors that signal through PI3K and AKT (e.g., *IGF1, HER2*) [1]. This signaling cascade therefore has received considerable attention in drug targeting, but balancing efficacy with safety (i.e., the therapeutic index) has proved to be a considerable hurdle to overcome [2]. Efforts directed at downregulating this pathway have focused largely on inhibiting protein function through small molecule inhibitors rather than on investigating potential mechanisms for silencing PI3-kinase/AKT signaling through transcriptional downregulation.

Similar to the PI3 kinase, *AKT1* is generally considered a ubiquitously expressed gene, and sequencing studies performed more than 20 years ago revealed that the *AKT1* locus contains GC-rich regulatory elements characteristic of housekeeping gene promoters [3]. We recently identified a novel *Ak11* transcript (*Akt1m*) that is controlled by a previously unknown mammary-specific promoter in mice [4]. The new transcript includes a completely different 3′ untranslated exon and encodes the full-length AKT1 protein with the ATG translation initiation codon in exon 2. Expression of *Akt1m* mRNA from this promoter is controlled by prolactin and JAK2/STAT5 signaling and is upregulated more than 500-fold during lactation compared to the virgin mammary gland, contributing to more than a 7-fold increase in total *Akt1* mRNA. The identification of this growth factor-induced promoter in mice provides a mechanism by which the levels of AKT1 can be temporally and spatially regulated at particular physiological states where heightened AKT1 activity is required (e.g., during lactation when metabolic needs are high).

It is an established fact that neoplastic cells hijack normal developmental pathways to support their unique metabolic requirements and to enhance cell proliferation, survival, and migration [5]. Using human cell lines and genetically engineered mice that are deficient in AKT1, it has been demonstrated that signaling through this serine-threonine kinase is critical for the initiation and progression of breast cancer [6-8]. Since growth factors such as prolactin and their downstream effectors play key roles in mammary tumorigenesis [9,10], it is feasible to hypothesize that cancer cells aberrantly activate the newly identified promoter to upregulate the transcriptional expression of *Akt1.* Given the histological and functional similarities of the mammary epithelium as well as the requirement of identical molecular pathways for the development of mammary glands in humans and mice, we postulated that the human genome might also contain an orthologous promoter that contributes to the transcriptional regulation of the *AKT1* gene. If this is the case, these orthologous regulatory elements might also be atypically activated in human breast cancers. This line of investigation might provide insight into the development of alternative strategies to modulate the expression of AKT1 in neoplastic cells.

## Methods

### Genetically modified mouse strains

The generation and analysis of the MMTV-Cre-based BRCA1 conditional knockout model (*Brca1*^*-/-*^) was described previously [11,12]. Mutant *Pten*^*G129E*^ mice [13] were kindly provided by Dr. Gustavo Leone (The Ohio State University). MMTV-neu transgenic mice [14] were obtained from the Jackson Laboratory. Transgenic lines that overexpress PRL in the mammary gland under the control of the neu-related lipocalin promoter [NRL-PRL] were published previously [15]. Mammary tumors that arose spontaneously in aging females of these genetically engineered mouse strains were flash frozen and stored in liquid nitrogen. All animals used in this study were treated humanely and in accordance with institutional guidelines and federal regulations. This study was carried out in strict accordance with the recommendations in the Guide for the Care and Use of Laboratory Animals of the National Institutes of Health. The protocol was approved by the Institutional Animal Care and Use Committee of the University of Nebraska Medical Center (IACUC#: 09-104-01, 03-104-01, and 12-008-03).

### Human breast cancer cell lines and tissue specimens

A panel of 43 human breast cancer cell lines was obtained from the American Type Culture Collection (ATCC) with financial support from the Integrative Cancer Biology Program at the National Cancer Institute (NCI). Forty-one of these cell lines were expanded and maintained using media and supplements recommended by ATCC. Deidentified flash-frozen human specimens representing normal tissues of the breast, lung, liver, pancreas, and stomach as well as nine human breast cancers representing the three major breast cancer subtypes (ERα-positive, ERBB2/HER2-positive, and triple-negative) were obtained under institutional guidelines from the tissue bank at the University of Nebraska Medical Center (UNMC).

### mRNA expression analyses using quantitative real-time PCR

Total RNA was extracted from flash-frozen tissues and cell pellets using standard guanidinium thiocynate-phenol-chloroform extraction or the RNeasy Mini Kit (Qiagen). The Super-Script II kit from Invitrogen with oligo-dT primers was used to perform the first-strand synthesis according to the manufacture’s protocol. Quantitative real-time PCR (qPCR) was performed using iQ SYBR green Supermix (Bio-Rad, Hercules, CA) and mRNA-specific forward primers for the mouse *Akt1m* (5′-GTC GCC ACC TGC TTG CTG AGG-3′) and the human orthologous *AKT1m* (5′-CCT TCC TCG AGT CTG GCC TG-3′). The reverse primers bind within the second coding exon of the mouse (5′-GGA CTC TCG CTG ATC CAC ATC C-3′) and the human *AKT1* (5′-GTA GCC AAT GAA GGT GCC ATC-3′) cDNAs, respectively. The qPCR reactions were carried out in triplicate in a CFX96 Real-Time PCR Detection System (Bio-Rad). The expression values obtained were normalized against *Gapdh* as described previously [4].

### Western blot analysis

Detailed experimental procedures for immunoprecipitation (IP) and western blot analysis were described elsewhere [16]. The following antibodies were used for immunoblotting: α-β-ACTIN (I-19) from Santa Cruz Biotechnology; α-pAKT (Ser473) (9271) from Cell Signaling and α-AKT1 (1081–1) from Epitomics.

### *In Silico* analysis

*In silico* genomic analyses of the human *AKT1* locus were performed using GenBank (http://www.ncbi.nlm.nih.gov/genbank/) and the UCSC Genome Browser (http://genome.ucsc.edu) [17,18]. The comprehensive analysis of the *AKT1* locus included assessing transcript variants, promoter genetic elements, interspecies genetic conservation, and reported ChIP data. The sequences of the mouse and novel human *AKT1m* cDNAs were submitted to GenBank (accession numbers KF836746 through KF836750).

### Statistical analysis

All graphic illustrations and statistics were performed with Prism 5 software (GraphPad Software, Inc., La Jolla, CA). Data are expressed as mean ± SD unless otherwise indicated and were compared using an unpaired Student *t* test. A *P* of less than .05 was considered significant.

## Results

### *Akt1m* is expressed and upregulated in the majority of mouse mammary tumors

**Figure 1:**
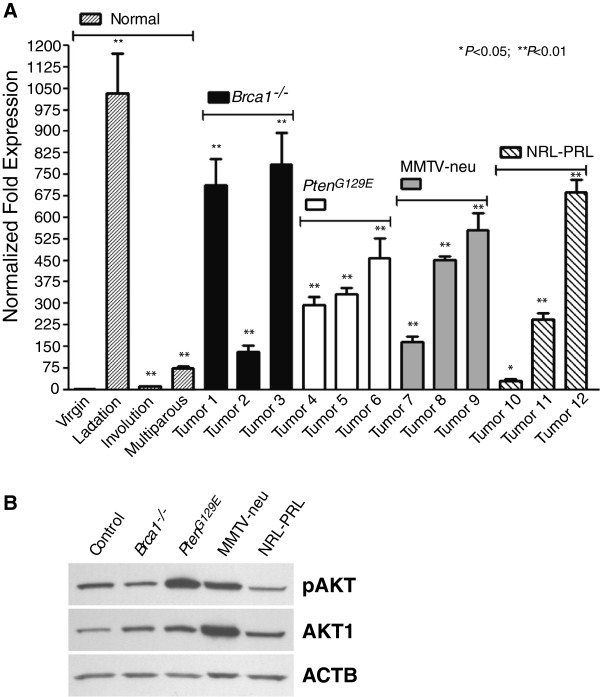
**The *Akt1m *transcript is upregulated during lactation and in mouse mammary tumors. (A)** Quantitative real-time RT-PCR analysis to assess the relative transcriptional activation of the *Akt1m* transcript at various stages of normal mammary gland development (nulliparous/virgin, lactation, involution, and non-pregnant multiparous) as well as 12 mammary tumors from Brca1 conditional knockout mice (MMTV-Cre *Brca1*^*fl/fl*^, tumors 1–3), a model for Cowden Syndrome (*Pten*^*G129E*^, tumors 4–6), and transgenic females expressing wildtype ErbB2 (*MMTV-neu*, tumors 7–9) or prolactin (*NRL-PRL*, tumors 10–12). Each sample was examined in triplicate and normalized to *Gapdh* expression. The relative expression levels of *Akt1m* were compared to the virgin control female, and statistical analysis was performed for each sample relative to the virgin control; *t* test, *P < 0.05, **P < 0.01. **(B)** Western blot analysis to determine the levels of phosphorylated AKT1 (pAKT, Ser473) and total AKT1 in selected mammary tumors (corresponding to tumors 1, 4, 7, and 10 in panel A) in comparison to a normal mammary gland of a multiparous female (control). Beta-actin (ACTB) was used as a loading control.

We performed quantitative RT-PCR on a panel of mammary tumors from diverse genetic cancer models to assess whether the *Akt1m* mRNA transcript is aberrantly expressed during mammary tumorigenesis in genetically engineered mice. Specifically, we examined the expression of *Akt1m* in primary cancers from BRCA1 conditional knockout mice (*Brca1*^*-/-*^), *Pten*^*G129E*^ mutant females as well as transgenic mice that overexpress ERBB2 (MMTV-neu) and prolactin (NRL-PRL). These models represent the major breast cancer subtypes found in humans, including triple-negative, basal-type lesions lacking BRCA1, HER2/ERBB2-positive tumors, as well as ERα-negative and ERα-positive, luminal-type cancers that originate in mice expressing mutant PTEN and prolactin in the mammary gland. The levels of expression in these neoplasms were matched to normal mammary gland tissues from virgin, lactating, involuting, and nonpregnant multiparous females. Consistent with our previous findings, *Akt1m* was upregulated approximately 1000-fold during lactation as compared to the virgin state. Its expression swiftly declined within two days following the weaning of the offspring and prior to postlactational remodeling of the mammary gland (Figure 1A). There was a small but noticeable increase in the expression of *Akt1m* in the multiparous mammary tissue. This is likely due to the emergence and expansion of a unique epithelial subtype, which we identified using genetic cell-fate mapping and named pregnancy-induced mammary epithelial cells (PI-MECs) [19,20]. These cells are prolactin responsive and located at the terminal ends of the ductal tree. They serve as alveolar progenitors during successive pregnancies, and we have previously demonstrated that they are the cells of origin for many MMTV-neu-induced mammary tumors [21]. In support of this notion, all mammary tumors from MMTV-neu transgenic females exhibited an elevated expression of the unique *Akt1m* transcript (Figure 1A). However, the vast majority of primary mammary cancers in all cancer models exhibited a significant increase in the expression of *Akt1m* regardless of the cellular subtypes that gave rise to luminal- or basal-type mammary tumors. *Pten*^*G129E*^ mutant mice were maintained as nulliparous females. Based on the significantly elevated expression of *Akt1m* in these tumors, it is evident that a full-term pregnancy and gestation cycle seems not to be a prerequisite for the upregulation of the unique AKT1 transcript in neoplastic mammary epithelial cells. As expected, the total levels of the AKT1 protein are elevated in all mammary tumor subtypes, but the activation of this kinase varies significantly and is highest in tumors with a known hyperactivation of the PI3 kinase in response to ERBB2 overexpression or loss-of-function of PTEN (Figure 1B). Efforts to elucidate the specific contribution of the *Akt1m* to the total pool of *Akt1* mRNA transcripts using qRT-PCR failed due to the high GC-rich content of the untranslated 5′exon following the basal promoter.

### The human genome contains a DNA sequence that is orthologous to the murine *Akt1m* and that gives rise to several new transcript variants encoding full-length AKT1

**Figure 2:**
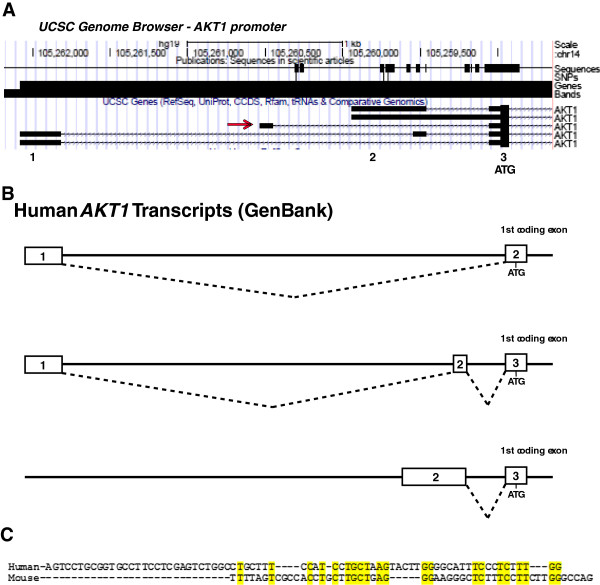
**Analysis of the human *AKT1 *locus and known transcripts to identify a sequence that is orthologous to the mouse *Akt1m*. (A)** Diagram of the human *AKT1* promoter region with transcriptional start sites and the location of 5′ untranslated exons of five currently known *AKT1* transcripts using the UCSC Genome Browser. The red arrow indicates an exon that is not associated with any of the known transcripts in GenBank. **(B)** Graphical illustration of all transcript variants of the human *AKT1* that are present in GenBank. **(C)** Genomic sequence comparison of the previously identified murine *Akt1m* exon with a putative human *AKT1m* ortholog according to the UCSC Genome Browser. Conserved nucleotides are highlighted in yellow (40% sequence similarity).

**Figure 3:**
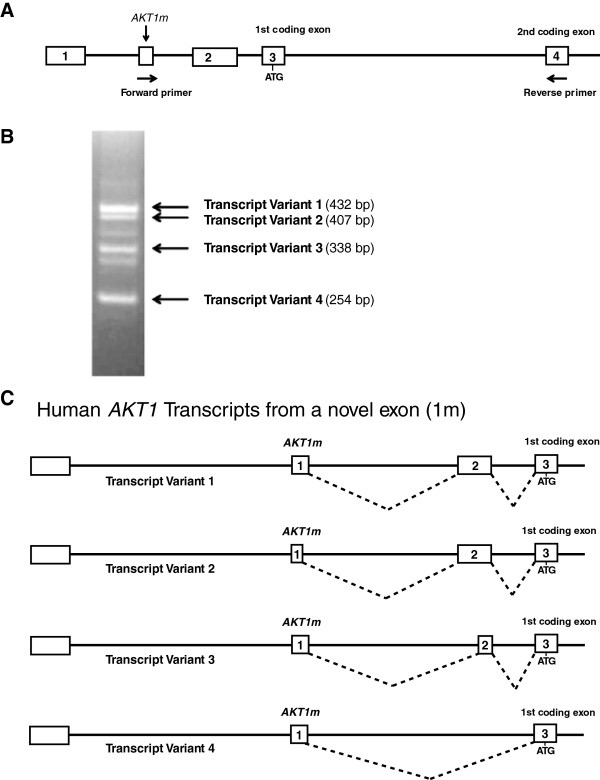
**Cloning of four novel human *AKT1 *transcripts that are orthologous to the mouse *Akt1m *mRNA. (A)** Schematic outline of the 5′ region of the human *AKT1* gene with the two currently known untranslated exons (1, 2) and the location of a novel *AKT1m* exon according to sequence comparison between the mouse and human genome. Arrows indicate the forward primer within *AKT1m* and the reverse primer in the second coding exon (i.e., exon 4) that were used to clone novel mRNA variants that originate from an alternative genomic region. **(B)** PCR amplicons of cDNAs from human T47-D breast cancer cells that contain the novel *AKT1m* exon. The four strongest bands were cloned and the presence of the *AKT1m* exon was confirmed by sequencing. The sizes of the sequenced amplicons are shown in parentheses. **(C)** Graphic illustration of the four new human *AKT1m* transcript variants that originate from a previously unidentified exon. Note that all of these transcripts splice into the first coding exon, suggesting that all new transcripts encode the full-length AKT1 kinase.

In an effort to identify the human *Akt1m* ortholog, we performed an *in silico* analysis of the genomic organization of the human *AKT1* gene and transcripts using the UCSC Genome Browser (Figure 2A). Four of the five *AKT1* gene transcripts that were listed resembled the three known sequences that were present in GenBank (Figure 2B). Interestingly, the fifth transcript arose from an untranslated exon that did not resemble any of the known GenBank entries and, furthermore, was located at a similar position relative to the ATG start site as the most 5′ murine *Akt1m* exon (Figure 2A, arrow). A comparison of the sequences of this particular exon with the mouse *Akt1m* DNA revealed they were, in fact, orthologous to one another and exhibit 40% sequence similarity within their overlapping region (Figure 2C). Based on the human sequence of the orthologous *Akt1m*, we designed a forward primer that binds specifically to this exon and a reverse primer that anneals within the second coding exon (i.e., exon 3). A PCR assay using these primers would allow detection of any *AKT1* mRNA transcripts in human cells that include this novel exon (Figure 3A). In contrast to the murine *Akt1m* exon, which produced only a single transcript in normal and neoplastic mammary epithelial cells, the PCR amplification of the human *AKT1m* produced multiple bands of varying sizes in T47-D human breast cancer cells (Figure 3B). The four strongest bands, denoted ‘Transcript Variants 1-4′, were gel purified, cloned, and sequenced. The sequence analysis confirmed that these novel *AKT1* transcripts contained the orthologous *AKT1m* exon depicted on the UCSC Genome Browser. Specifically, the *AKT1m* transcript that we identified using *in silico* analysis represented the transcript variant 4 (Figure 3C). Additionally, we cloned three new mRNA variants consisting of the same initial 5′ untranslated exon or a shortened version of the exon in the case of transcript variant 2. These longer transcript variants spliced into a second untranslated exon in front of the first coding exon containing the ATG start site of the full-length AKT1. Only the second exon is also shared with a transcript that originates from the GC-rich basal promoter (Figure 2B, middle). Collectively, the results from the cloning and sequencing analysis of novel *Akt1* mRNA variants reveal that the transcriptional activation of this gene locus is more complex than previously thought. Results from our own 5′RACE experiments [4] and the analysis of all published sequences in GenBank showed that the *Akt1m* non-coding exon was never included in any of the mRNAs that originated from the basal promoter. All previously and newly identified transcripts utilize the same first coding exon containing the ATG translation initiation codon and splice correctly into the following coding exons. It is therefore evident that, like in mice, the full-length AKT1 kinase is encoded by messenger RNAs from at least two distinct promoters within the *AKT1* locus.

### A highly conserved sequence immediately upstream of the *AKT1m* exon serves as a second promoter within the *AKT1* locus

**Figure 4:**
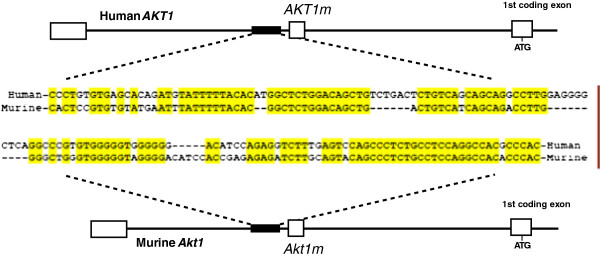
**Identification of a conserved putative promoter sequence immediately upstream of the novel human and murine *AKT1m *exon.** The black bar within the graphic illustrations of the 5′ regions of the human and mouse *AKT1* loci represents a highly conserved genomic region of 158 bps in length. This region is located approximately 60 and 90 bps upstream of the human and mouse *AKT1m* exon, respectively. The sequences exhibited a 69% conservation (bases highlighted in yellow).

Using the UCSC Genome Browser, we performed another *in silico* analysis to identify regions of high similarity between the human and mouse sequences preceding the *Akt1m* exon. The presence of conserved sequences might indicate the existence of a unique promoter that drives the expression of the alternative *Akt1* mRNA encoding AKT1. This analysis revealed a DNA sequence of 158 bps in length that exhibits a 69% conservation between mouse and human beginning approximately 60 bps upstream of *Akt1m* (Figure 4). Only 31% of the nucleotides are similar in the sequence preceding the conserved region, and the DNA exhibits merely 14% conservation within intron 1 following the *Akt1m*. Interestingly, the high-probability STAT5 binding site [TTC(T/C)N(G/A)GAA [22]] downstream of exon 1 that we identified and verified using ChIP analysis in mouse tissues is missing in the human genome, suggesting that prolactin signaling may not be directly involved in the transcriptional regulation of the expression of *AKT1m* in humans. However, the existence of a highly conserved region in close proximity of the transcriptional start site of *Akt1m* is suggestive of a putative promoter, and a subsequent analysis performed using the Transcription Element Search System (TESS, University of Pennsylvania) revealed multiple conserved binding sites for transcriptional regulators such as Activator Protein-1 (AP-1) and the glucocorticoid receptor (GR). Additional *in silico* analyses of whole genome ChIP sequencing (ChIP-Seq) data using the UCSC Genome Browser showed that RNA polymerase II and the c-MYC proto-oncogene are bound to the conserved region preceding the *AKT1m* exon in MCF7 breast cancer cells [17,18].

### Human *AKT1m* transcripts are expressed and upregulated in a subset of breast cancer cell lines and primary human breast cancer specimens

**Figure 5:**
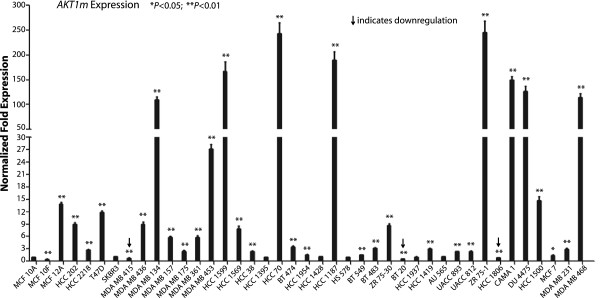
***AKT1m *transcripts are expressed and upregulated in a subset of human breast cancer cell lines.** Quantitative real-time RT-PCR analysis to assess the relative transcriptional activation of the *AKT1m* transcripts in a panel of three untransformed human breast epithelial cell lines and 38 breast cancer cell lines that represent all major subtypes of this malignancy (i.e., ERα-positive, ErbB2-positive, and triple-negative). Each sample was examined in triplicate and normalized to *GAPDH* expression. The expression of *AKT1m* in untransformed MCF-10A cells was set to 1.0 and statistical analysis was performed for each cell line relative to the MCF-10A control; *t* test *P < 0.05, **P < 0.01.

The cloning of *AKT1m* mRNA transcripts from T47-D breast cancer cells and the binding of RNA polymerase II and c-MYC to conserved sequences immediately upstream of the start site of these novel mRNA variants encoding AKT1 are evidence for the expression of *AKT1m* in neoplastic mammary epithelial cells. To verify the presence of the transcripts and to determine the levels of *AKT1m* mRNA expression, we performed a quantitative RT-PCR assay across a panel of 41 human breast epithelial cell lines. The panel consisted of three non-tumorigenic lines (MCF-10A, MCF-10F, and MCF-12A) and 38 selected cancer cell lines from ATCC that represent all major human breast cancer subtypes (Figure 5). The relative expression of *AKT1m* was normalized against *GAPDH* and the expression level of this mRNA in MCF-10A cells. With the exception of MCF-12A cells, the expression of *AKT1m* was significantly lower in immortalized, untransformed mammary epithelial cells (MCF-10A and MCF-10F) compared to the majority of breast cancer cells. Nearly a quarter (24%) of the 38 breast cancer samples expressed *AKT1m* transcripts at levels at least two-fold greater than all three non-tumorigenic samples, and 33 of 38 lines (87%) exhibited expression levels that were higher than both MCF-10A and MCF-10F cells. Collectively, 29 of the 38 breast cancer cell lines displayed significantly greater *AKT1m* expression compared to MCF-10A control cells. Six lines lacked statistical significance, and three breast cancer cell lines exhibited lower expression. Similar to mouse mammary tumors, there seems to be no obvious correlation between *AKT1m* upregulation, hormone receptor status, or breast cancer subtype.

**Figure 6:**
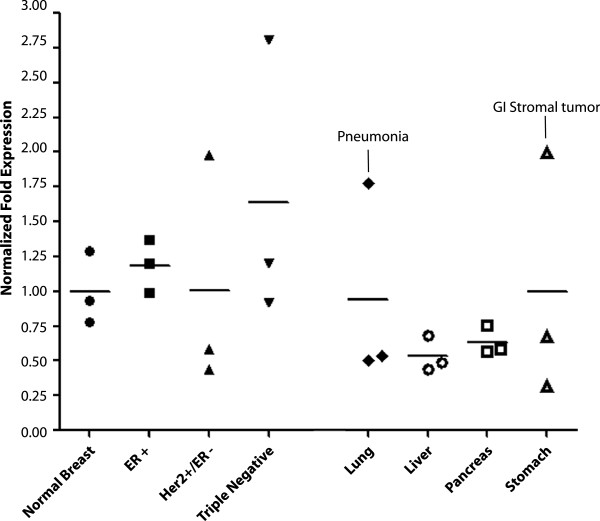
**Expression of *AKT1m *in normal breast tissues and selected breast cancer specimens.** qRT-PCR analysis to determine the relative expression of the *AKT1m* transcript variants in three primary human breast tissues and nine breast cancers from different patients, including three ERα-positive as well as three Her2-positive/ERα-negative and three triple-negative specimens. Additional samples from other organs (i.e., lung, liver, pancreas, and stomach) were included into this analysis to assess a possible widespread or tissue-specific expression of the *AKT1m* transcripts. Note there was a clear elevation in the expression of *AKT1m* in the lung and stomach of two patients who were diagnosed with pneumonia and a GI stromal tumor, respectively (circles). Each sample was examined in triplicate in the qPCR assay and normalized to *GAPDH* expression; bars represent SEM.

Next, we verified the transcriptional activation of *AKT1m* using quantitative RT-PCR on three primary human breast tissues and nine breast cancers, including three ERα-positive, three HER2-positive/ERα-negative, and three triple-negative specimens (Figure 6). We also included additional samples from other organs into this analysis (i.e., lung, liver, pancreas, and stomach) to assess whether, similar to the expression profile that we observed in mice, *AKT1m* expression is largely confined to the mammary gland. While the *AKT1m* transcripts were detectable in the normal human breast derived from reduction mammoplasties, there was only a marginal increase in the transcriptional activation in ERα-positive tumors. Unlike in genetically defined lesions of the mouse mammary tumor models that exhibited elevated levels of *Akt1m* in virtually all tumors, the expression of the orthologous transcripts in human breast cancers were more variable and therefore lacked statistical significance. However, some of the ERα-negative or triple-negative breast cancer tissues exhibited a much greater expression of *AKT1m* than all three normal breast specimens. The data from this analysis on a limited number of primary tumor samples of the three major breast cancer subtypes essentially mirrors the quantitative RT-PCR results performed on a more extensive and well annotated panel of breast cancer cell lines. Also, the expression of *AKT1m* was lower in other healthy organs compared to the normal mammary gland (Figure 6). Interestingly, two samples in the lung and stomach showed a clear elevation in the expression of *AKT1m* in patients that were diagnosed with pneumonia and a gastric stromal tumor. Collectively, the results from the analysis of normal and neoplastic cells and primary tissues indicate that, unlike in mice, the transcriptional activation of *AKT1m* is not entirely confined to the mammary gland. However, the expression of *AKT1m* transcripts is substantially higher in a subset of breast cancer cell lines as well as primary human breast cancers, and perhaps, in malignancies or pathological conditions in organs other than the mammary gland.

## Discussion

The AKT serine-threonine protein kinases exhibit a wide-spread expression pattern in virtually all human cell types. They are activated downstream of various growth factor receptors, in particular by receptor tyrosine kinases, through PI3 kinase-dependent mechanisms. The three AKT isoforms (AKT1-3) control a number of intracellular processes such as growth, proliferation, metabolism, and cell survival [23-25]. Studies in single, double, and triple knockout mice have shown that the three AKT proteins can have redundant and non-redundant functions in particular cell types [26-28]. Among the three AKT members, only AKT1 has been shown to be crucial for normal mammary gland development. During pregnancy and lactation, this kinase is upregulated in the mammary epithelium where it controls metabolic pathways that regulate milk synthesis and the functional differentiation of the gland [27,29]. Immediately following the cessation of lactation and weaning of the offspring, AKT1 mRNA and protein levels decline rapidly to facilitate a swift remodeling of the mammary epithelium [4,30]. A sustained expression of hyperactive or wildtype AKT1 is entirely sufficient to delay apoptosis and mammary gland involution [30-32]. The expression and functionality of AKT1 parallels closely the biological functions of prolactin and its downstream signaling mediators. We have demonstrated previously that the activation of AKT1 as well as the total levels of this kinase are dependent on the Janus kinase 2 (JAK2) and active STAT5 [16]. More recently, we identified a novel role of JAK2/STAT5 signaling in the transcriptional activation of the *Akt1* gene in mice [4]. Upon binding to the promoter of *Akt1* in a growth factor-dependent manner, STAT5 initiates the transcription of a unique *Akt1* mRNA from a distinct promoter, which was only present in the mammary gland. Using transgenic mice that express hyperactive STAT5 in a ligand-regulatible manner, we demonstrated that gain-of-function of this transcription factor mediates a sustained upregulation of *Akt1 in vivo*[4]. Phenotypically similar to females that overexpress AKT1, the prolonged activation of STAT5 impaired postlactational remodeling of the mammary gland. Collectively, the results of our previous lines of investigation revealed a novel mechanism by which the *Akt1* gene can be transcriptionally regulated from an alternative promoter depending on the developmental state and physiological needs.

Active STAT5 and AKT1 both mediate evasion from apoptosis and self-sufficiency in growth signals, which are hallmarks of cancer. In support of this notion it has been observed that both signal transducers exhibit a deregulated expression and activation in human breast cancers. Moreover, it has been demonstrated that JAK2/STAT5 signaling and AKT1 play essential roles during mammary tumor initiation in various murine cancer models [7,8,33-36]. Specifically, upregulation and activation of AKT1 is required to sustain a hypermetabolic state (e.g., “Warburg effect”) that is a unique characteristic of certain cancer cells [37,38]. As demonstrated in this report, the majority of luminal- or basal-type mammary tumors showed an increased expression of AKT1 on the protein level and a significant upregulation of the *Akt1m* transcript. Similar to the regulation of AKT1 during normal mammary gland development, cancer cells are able to upregulate this serine-threonine kinase on the transcriptional level to meet the specific metabolic needs in the transformed state. Using *in silico* analysis, we were able to identify the human ortholog of the murine *Akt1m,* but unlike in mice that only express a single *Akt1m* mRNA, we cloned four new transcripts in human cells that originated from a previously unidentified, alternative promoter. Since the ATG start codon is located within the downstream exons (i.e., exons 2 or 3 depending on the specific transcript variants), it is evident that all four newly identified mRNAs include the first coding exon and therefore encode the full-length AKT1 kinase. RT-PCR results using the *AKT1m* specific primer in the 5′UTR in combination with two reverse primers within downstream coding exons confirmed a correct splicing within the CDS of the *AKT1* mRNA. The four new *AKT1m* transcripts were initially cloned from prolactin-responsive T47-D breast cancer cells, but the analyses of a larger panel of breast cancer cells as well as primary tumors show that expression of *AKT1m* is not restricted to luminal-type cancer cells. Although their levels are lower, the *AKT1m* transcript variants were also detectable in untransformed mammary epithelial cells and normal breast tissue specimens. This suggests that they are not a result of aberrant splicing, which more frequently occurs in transformed cells [39]. Another distinct characteristic is the presence of *AKT1m* in other normal human tissues (i.e., lung, liver, pancreas, and stomach). Although the expression in these organs was typically lower compared to the breast, the *Akt1m* was not detected at all in tissues other than the mammary gland in mice using RT-PCR. The notion that the regulation of the alternative *AKT1m* promoter might show some species-specific differences is supported by the absence of the STAT5 binding sites in the human locus. In mice, we found two high-probability STAT5 binding sites upstream and immediately downstream of the *Akt1m* exon. Using chromatin immunoprecipitation (ChIP) and quantitative PCR on lysates from cultured cells as well as mammary gland tissues, we demonstrated that STAT5 binds to these particular recognition sites in a growth factor-dependent manner to significantly enhance *Akt1m* transcription [4]. Like the activation of milk protein gene promoters, STAT5 seems to confer a tissue-specific expression profile of *Akt1m* in conjunction with other transcription factors such as the glucocorticoid receptor in the mouse [40]. The absence of STAT5 binding sites might account for the lack of mammary gland specificity in humans. However, there are multiple GR binding sites present within the highly conserved orthologous region in the human sequence immediately upstream of the first exon of *AKT1m*. More importantly, the confirmed presence of c-MYC on the conserved putative promoter sequences immediately upstream of *AKT1m* using ChIP might be indicative of a growth-factor controlled expression of AKT1 depending on metabolic needs. In support of this notion, both AKT1 and c-MYC synergistically promote metabolic reprograming and aerobic glycolysis in cancer cells [37,38]. The identification of a novel putative promoter sequence in *AKT1* that is conserved between humans and mice is an interesting finding, but additional work is required to further pinpoint and confirm the functionality of the *AKT1m* promoter along with the transcription factors that control its activation.

Regardless of the species-specific nuances in the regulation of *Akt1m*, it is evident that the transcriptional regulation of the *Akt1* locus is more complex than previously thought. In mice as well as humans, the *Akt1* gene is transcriptionally upregulated in a subset of cancer cells, and the growth factor-dependent activation activation of this locus occurs through at least two distinct promoters. This is supported by our 5′RACE data and published sequences in GenBank that the *AKT1m*-specific untranslated exon is absent in other known mRNA sequences that start at the originally identified promoter located much further upstream [3]. The latter promoter and associated non-coding exon are very GC-rich, and attempts to generate primer sets to discriminate and to quantify the contribution of the *AKT1m* transcripts to the total pool of *AKT1* mRNA messages have been unsuccessful. Interestingly, besides the upregulation of *AKT1m* in a subset of human breast cancers, elevated levels of these transcript variants were also found in other diseased tissues (i.e., pneumonia of the lung, and GI stromal tumor). The qRT-PCR assay that we employed might be a simple, yet sensitive, diagnostic tool to assess pathological changes indicative of an altered metabolism that may correlate with a transcriptional upregulation of *AKT1*.

## Conclusions

The collective results of this study suggest that, like in mice, the expression of the *AKT1* locus in humans is controlled by at least two distinct promoters, suggesting that the transcriptional activation of this gene is more complex than previously thought. Four novel transcript variants were identified in breast cancer cell lines that are orthologous to the mouse *Akt1m* mRNA message. All encode the full-length AKT1 serine threonine protein kinase, and these transcripts originate from a putative promoter sequence that is conserved between humans and mice. All mammary cancers that developed in diverse genetically engineered mouse models as well as a subset of human breast cancer cell lines and primary breast cancers exhibited a much higher expression of *AKT1m. AKT1* is generally viewed as a persistently active house-keeping gene, but the existence of an alternative promoter within this gene locus may provide a mechanism by which the levels of AKT1 can be temporally and spatially regulated at particular physiological states, such as cancer, where a heightened activity of this kinase is required. Further studies will show whether targeting specifically the expression of *AKT1m* is a suitable strategy to downregulate AKT1 in breast cancer cells without a complete ablation of the ubiquitously expressed transcripts in normal tissues.

## Abbreviations

5′RACE: Rapid amplification of cDNA ends at the 5 prime of the mRNA; AKT/PKB: Serine-threonine protein kinase; BRCA1: Breast cancer 1 susceptibility gene, early onset; ChIP: Chromatin immunoprecipitation; Cre: Site-specific recombinase; ERα: Estrogen receptor alpha; GAPDH: Glyceraldehyde 3-phosphate dehydrogenase, GR: Glucocorticoid receptor; JAK2: Janus kinase 2; MMTV: Mouse mammary tumor virus; neu: ERBB2: Receptor tyrosine kinase; NRL: Neu-related lipocalin; PI-MECs: Parity-induced mammary epithelial cells; PI3K: Phosphoinositide 3-kinase; PRL: Prolactin; PTEN: Phosphatase and tensin homolog; qPCR: Real-time quantitative polymerase chain reaction; SD: Standard deviation; STAT5: Signal transducer and activator of transcription 5.

## Competing interests

The authors declare they have no competing interests.

## Authors’ contributions

JWS and KUW designed the research, supervised all experiments, and drafted this paper. BLW executed RT-PCR experiments. KS and AAT assisted in animal experiments and collected mammary tumors. WWW performed the histopathology on breast cancer specimens and selected cases for particular breast cancer subtypes and normal tissues. All authors read and approved the final manuscript.

## Pre-publication history

The pre-publication history for this paper can be accessed here:

http://www.biomedcentral.com/1471-2407/14/195/prepub
